# Supplementary material for: Prevalence of Sexualized Substance Use and Chemsex in the General Population and Among Women: A Systematic Review and Meta-Analysis of Cross-Sectional Studies
Source: Healthcare (Basel). 2025 Apr 14;13(8):899. doi: 10.3390/healthcare13080899 (PMC12026793; doi:10.3390/healthcare13080899)
Supplement: Supplementary file 1 [file healthcare-13-00899-s001.zip › Figures S1-S3.pdf]

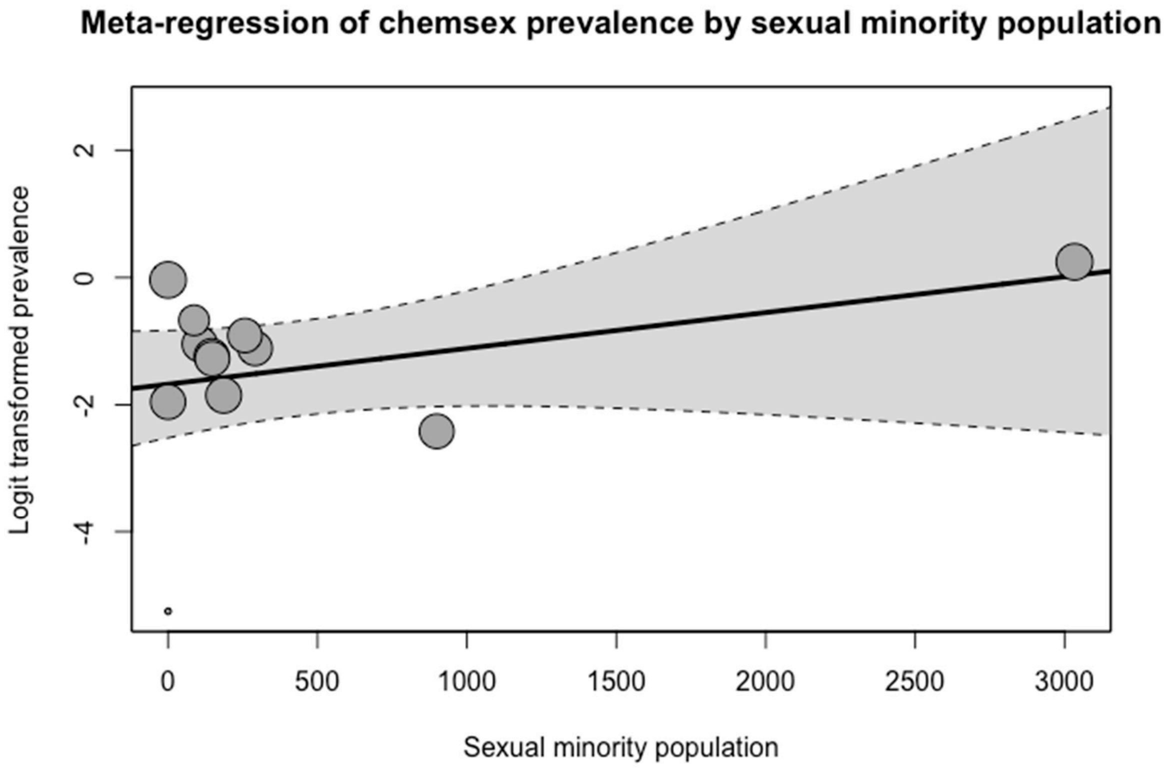

Figure S1. Meta-regression of chemsex prevalence by sexual minority population among the general popula-tion.

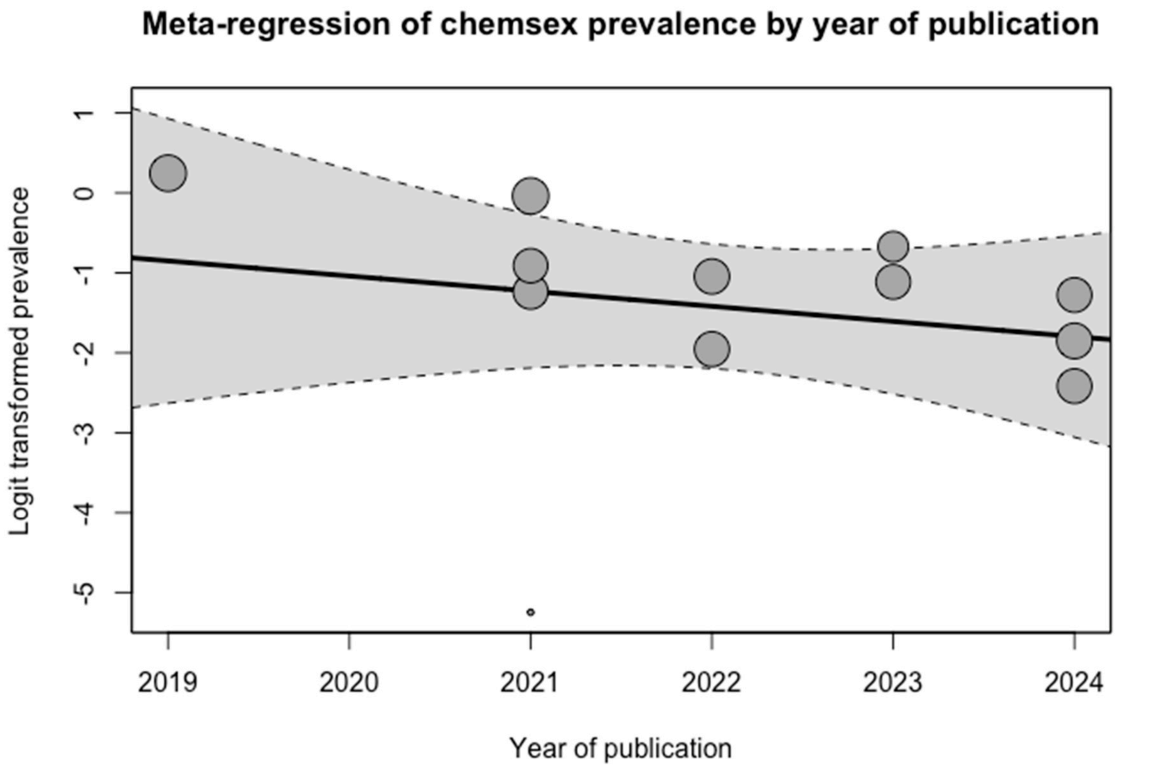

Figure S2. Meta-regression of chemsex prevalence by year of publication among the general population.

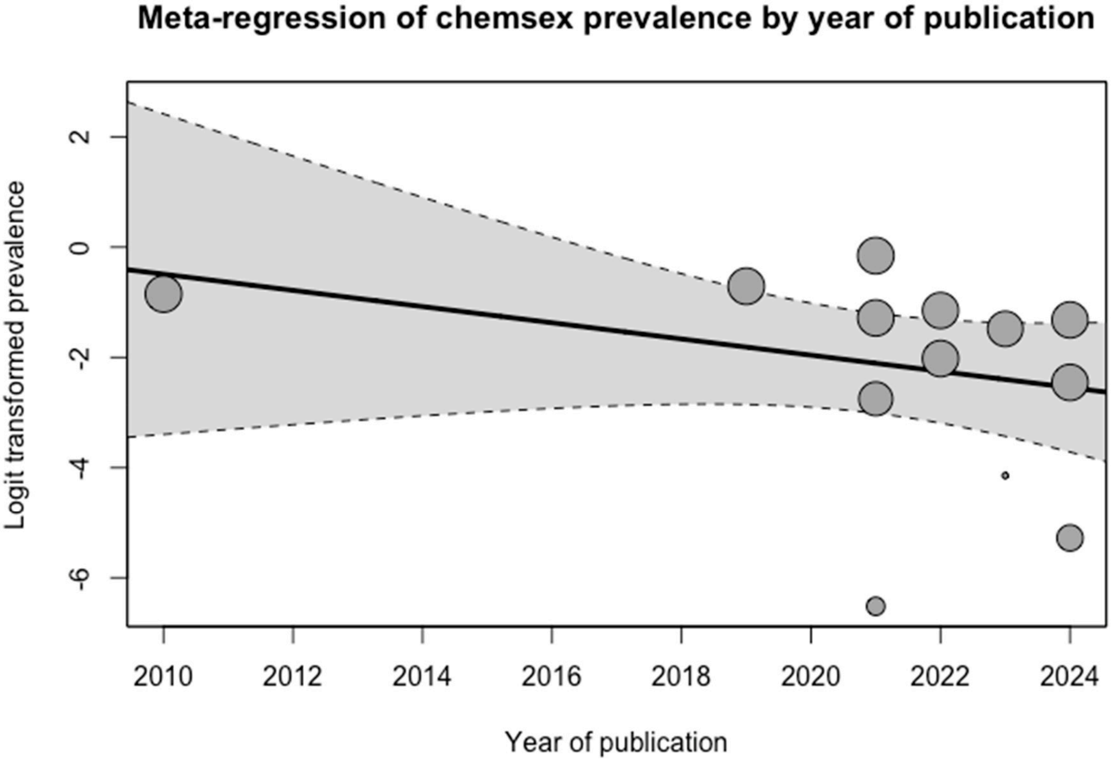

Figure S3. Meta-regression of chemsex prevalence by year of publication among the female population.
