# Supplementary figures and images for: Prevalence of Sexualized Substance Use and Chemsex in the General Population and Among Women: A Systematic Review and Meta-Analysis of Cross-Sectional Studies
Source: Healthcare (Basel). 2025 Apr 14;13(8):899. doi: 10.3390/healthcare13080899 (PMC12026793; doi:10.3390/healthcare13080899)

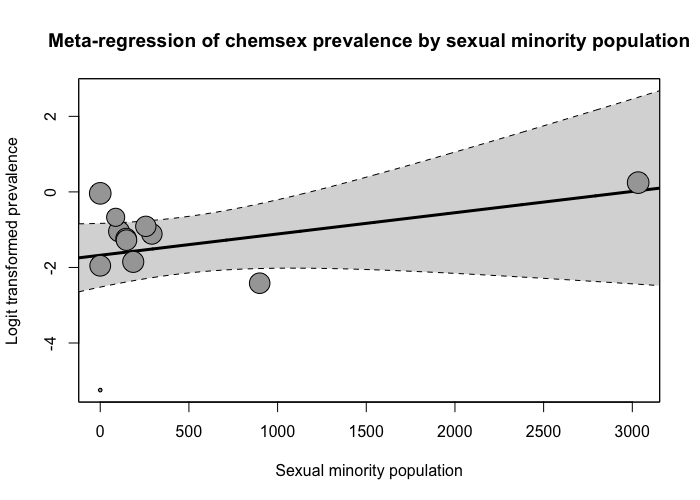

Supplement: Supplementary file 1 [file healthcare-13-00899-s001.zip › Figure S1.tiff]

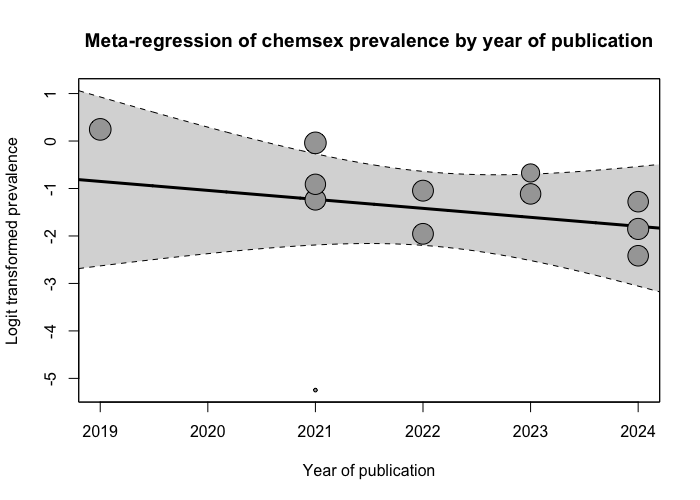

Supplement: Supplementary file 1 [file healthcare-13-00899-s001.zip › Figure S2.tiff]

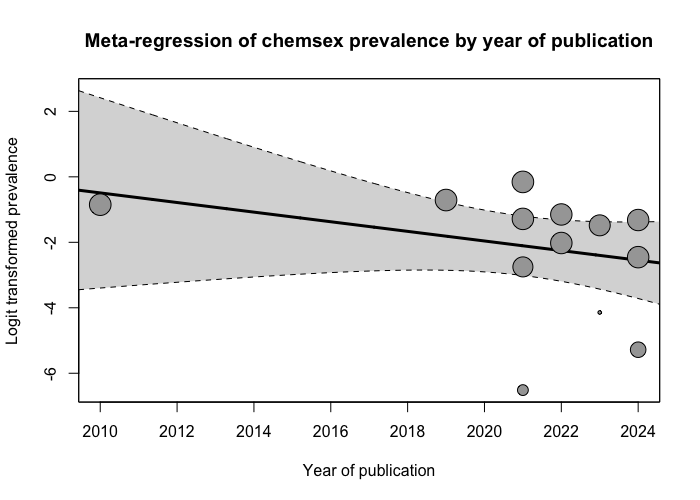

Supplement: Supplementary file 1 [file healthcare-13-00899-s001.zip › Figure S3.tiff]
